# Artificial rupture of membranes as a mode for induction of labor in women with a previous cesarean section- a retrospective cohort study

**DOI:** 10.1186/s12884-022-05237-2

**Published:** 2022-11-30

**Authors:** Aharon Dick, Einat Gutman-Ido, Henry Hillel Chill, Gilad Karavani, Ina Ryvkin, Shay Porat, Joshua Isaac Rosenbloom

**Affiliations:** 1 Department of Obstetrics and Gynecology, Hadassah Medical Center and Hebrew University of Jerusalem, Jerusalem, Israel; 2 Department of Obstetrics and Gynecology, Hadassah Medical Organization and Faculty of Medicine, Hebrew University of Jerusalem, Jerusalem, 91120 Israel; 3 Division of Urogynecology, University of Chicago Pritzker School of Medicine, NorthShore University HealthSystem, Skokie, IL USA

**Keywords:** Amniotomy, Artificial rupture of membranes (AROM), Induction of Labor (IOL), Oxytocin, Trial of labor after cesarean section (TOLAC), Vaginal birth after cesarean (VBAC)

## Abstract

**Background:**

Induction of labor in women with a previous cesarean section (CS) is associated with increased rates of uterine rupture and failed attempt for vaginal delivery. Prostaglandins use is contraindicated in this population, limiting available options for cervical ripening.

**Objective:**

To evaluate the efficacy and safety of artificial rupture of membranes (AROM) as a mode of Induction of labor (IOL) in women with a previous cesarean section.

**Methods:**

A retrospective cohort study conducted in a single tertiary care center between January 2015 and October 2020. Women with one previous cesarean section and a current singleton term pregnancy requiring IOL, with an unfavorable cervix, were included. The primary outcome was a successful vaginal delivery (VBAC); secondary outcomes were rates of chorioamnionitis, uterine rupture and low Apgar score (< 7).

**Results:**

Of the 665 women who met the inclusion criteria, 492 (74%) did not receive subsequent oxytocin and 173 (26%) did. There were significant differences in the baseline characteristics between these two groups, including maternal age, cervical dilation at presentation, parity, and a history of a previous VBAC. Among women who were induced solely by AROM the rate of a successful TOLAC was higher (81.3% vs 73.9%), total time of IOL was shorter (mean 8.7 h vs.16.1 h) and the risk of chorioamnionitis was lower (7.3% vs 18.4%). When subdividing the women who received oxytocin into early (< 12 h after AROM) vs late (> 12 h after AROM) administration, there were no significant changes in the rates of successful VBAC or of chorioamnionitis.

**Conclusion:**

AROM as a single mode of IOL in women with a previous CS is a safe and efficient practice with high rates of successful VBAC. When spontaneous labor does not develop, there is no advantage to delay the administration of oxytocin.

## Backround

Induction of labor (IOL) is a common obstetric practice with continues increasing rates: from 9.5% in 1990 up to 40% in 2012 [1, 2]. Current options for IOL include use of oxytocin, prostaglandins, artificial rupture of membranes (AROM), and balloon catheter placement. However, IOL in women with a previous cesarean section challenges the obstetric clinician due to increased risk of failed trial of labor (TOLAC) and uterine rupture [3–6]. Additionally, several other maternal morbidities such as need for blood transfusion, thromboembolism, and hysterectomy have been reported [7–9].

When evaluating the different options for IOL in women with a previous cesarean section, the clinician has limited options because prostaglandins are contraindicated due to the increased risk of uterine rupture [10–13]. Furthermore, although not contraindicated, use of oxytocin is also associated with an up to twofold increased risk of uterine rupture [3, 14, 15] as well as adverse neonatal outcomes [16].

Artificial rupture of membranes (AROM) is a non-pharmacological mode of IOL, with high success rates especially in multiparous women [17]. To date, little is known about the efficacy of AROM as a mode of IOL in women with a previous cesarean section. Therefore, the primary aim of this paper was to evaluate the safety and utility of AROM in this population. Secondly, we aimed to determine whether delayed administration of oxytocin is of benefit in comparison to early administration in women who did not enter active labor after AROM.

## Methods

This is a retrospective cohort study at large academic medical center of women with a history of one previous CS undergoing an IOL for TOLAC. All women with a viable singleton term (gestational age ≥ 37 weeks 0 days) pregnancy, cephalic presentation and a cervical dilation of < 4 cm, undergoing TOLAC between January 1 2015 and October 1 2020 were identified in the hospital electronic medical record.

Women with previous classical CS, history of more than one CS, active labor, pre-labor rupture of membranes, scheduled elective CS, preterm delivery, breech presentation, multi fetal pregnancy or fetal demise were excluded from the study.

Our primary outcome was the rate of successful vaginal delivery. Secondary outcomes were total time of IOL, rates of chorioamnionitis, uterine rupture and low Apgar score (< 7).

The default mode for IOL for patients undergoing TOLAC in our hospital is AROM. This is due to the increased risk of uterine rupture with the use of prostaglandins or oxytocin in this population. In the minority of cases (less than 10%) AROM is not possible. The main reasons for this situation are a long cervical length (membranes unreachable by vaginal examination) and a high head station which may be associated with cord prolapse [18]. In these situations, IOL was initiated with oxytocin prior to AROM, and these women were excluded from our study.

After AROM was performed, women were hospitalized to await a spontaneous onset of labor. Conservative management was limited to up to 48 h. At the onset of labor, patients were admitted to the labor ward. Women with signs suggesting chorioamnionitis such as fever, abdominal tenderness, foul-smelling vaginal discharge, leukocytosis, or maternal or fetal tachycardia were transferred immediately to the labor ward. Likewise, women with indications for immediate delivery such as preeclampsia or uncontrolled diabetes were not offered expectant management after AROM; instead, oxytocin was initiated shortly after AROM. In the reminder of cases, shared patient-physician decision making was used to decide on the timing of initiation of oxytocin.

Our protocol for oxytocin in women undergoing TOLAC recommends an initial dose of 1 miU/min. At 30-min intervals, the dose is gradually increased by increments of 1 miU/min until a maximum of 20miU/min or when a desired contraction pattern is established.

Maternal and neonatal data were retrieved using a computerized database, continuously updated and validated for admission, labor, and postpartum course.

Data collected included maternal age, maternal weight, gravidity, parity, history of previous vaginal delivery or VBAC. Data regarding current labor included gestational age (determined by early ultrasound), cervical dilatation at AROM, mode of delivery, oxytocin use, latency in minutes from AROM to oxytocin, total time of oxytocin administration and total induction time. Neonatal data included birthweight and 5-min APGAR score.

Initially, outcomes and rates of successful vaginal delivery were compered between AROM alone and AROM followed by oxytocin administration. Thereafter a second analysis was performed including only women who received oxytocin. Outcomes were compared between early (≤ 12 h) and late (> 12 h) oxytocin administration.

### Statistical analysis

Continuous variables with normal distribution were compared using the Students t-test. The continuous variables without normal distribution were compared using Mann–Whitney U test. Categorical variables were compared using Pearson chi-square.

Multivariable logistic regression was conducted to identify the variables that were significantly associated with a successful VBAC or with chorioamnionitis. A *p* value of < 0.05 was considered significant.

Statistical analysis was performed by using IBM SPSS Statistics for Windows, Version 24.0 (Armonk, NY, USA). The local Institutional Review Board approved the study protocol.

## Results

**Fig. 1:**
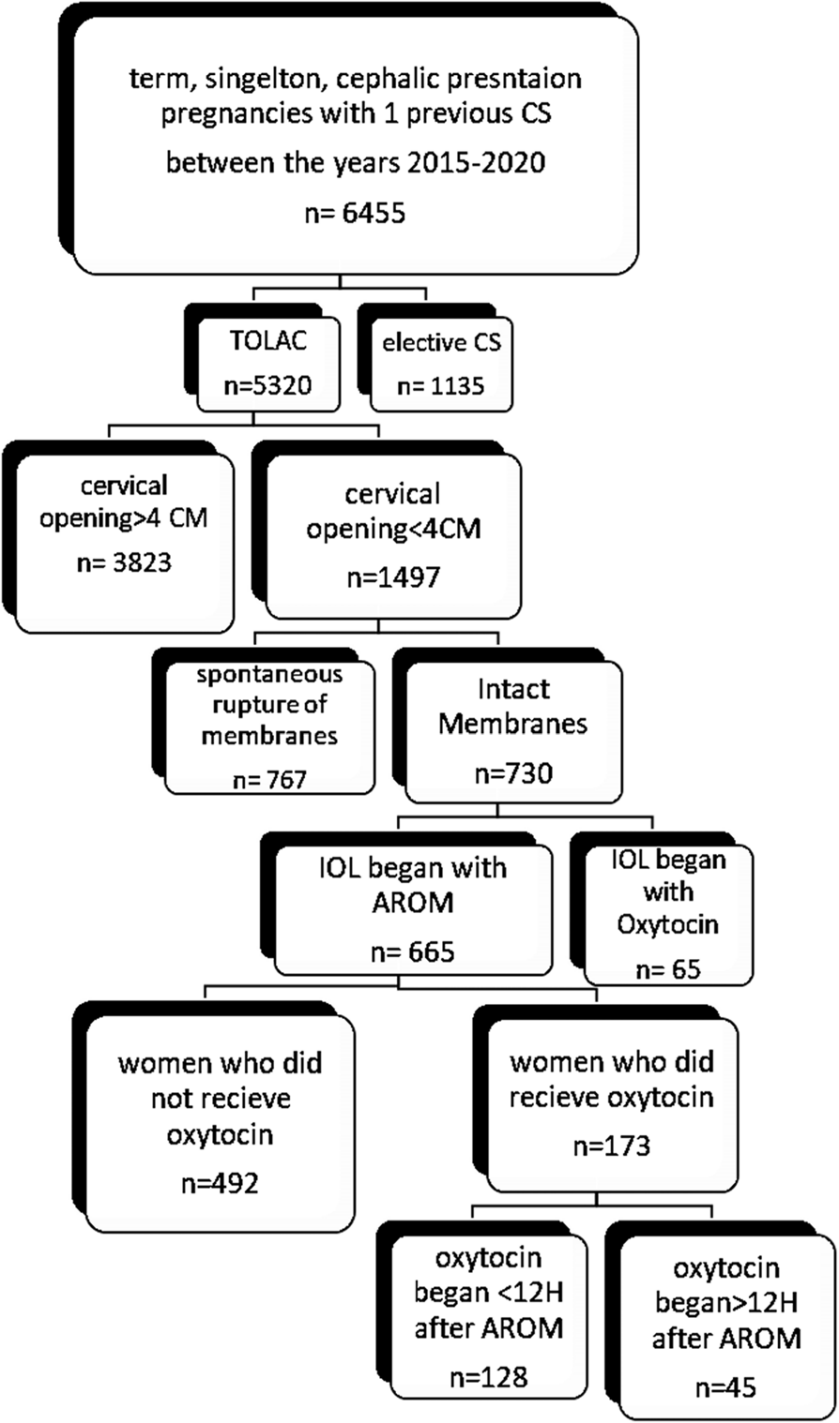
Flow chart of eligible women included in our study

During the study period 5320 women with one previous CS and a term, cephalic presentation pregnancy presented for TOLAC. 3823 were in active labor (defined as cervical dilation of 4 CM or more) and further 767 had spontaneous ROM, leaving 730 women with a cervical dilation of < 4 cm and intact membranes requiring an induction of labor (Fig. 1). Of them, oxytocin was used as the initial mode of induction in 65 women (9%), who were excluded from our study. Therefore, there were 665 women eligible for analysis. Of these 665 women induced by AROM, 492 (74%) did not require subsequent use of oxytocin 528 (79.39%) of patients had a successful vaginal delivery. Among those who did not receive oxytocin, the rate of a successful VBAC was significantly higher (81.3% vs.73.9% *p* = 0.03).

Maternal characteristics of the two groups are presented in Table 1. Significant differences were noted between the groups in maternal age, history of a previous VBAC, and parity. Cervical dilation median at presentation was 3 and 3.5 CM, respectively (*p* < 0.01).

**Table 1:** Baseline Characteristics for Women Who Received and did not Receive Oxytocin after AROM

	**No oxytocin (492)**	**Oxytocin (173)**	***P***
**Age, years**	33 (29–37)	31(27–35)	< 0.01
**Gestational age, weeks**	40.1(39.1–41)	40(39.1–40.5)	0.41
**Weight, kg**	76.0(15.1)	80.0(16.6)	0.45
**Cervical dilation at AROM, cm**	3.5 (3–4)	3(2–3.5)	< 0.01
**Previous VBAC**	209(42%)	56(32%)	0.01
**No of previous VBAC**	2 (1–3)	2(1–3)	0.96
**Parity**	2(1–4)	2(1–3)	< 0.01

Numbers are n (%) or median (interquartile range) or mean (standard deviation)

Of the 173 women who received subsequent oxytocin, 128 (74%) received it less than 12 h after AROM (early initiation) and 45 (26%) women received it after 12 h (late initiation). Maternal characteristics of these two groups are presented in Table 2. The two groups did not differ in the baseline characteristics except from the initial cervical dilation at AROM, which was significantly higher in the early initiation group.

**Table 2:** Baseline Characteristics of Early vs Delayed Administration of Oxytocin

	**Oxytocin started at < 12 h(128)**	**Oxytocin started at ≥ 12 h (45)**	***P***
**Age, years**	31(27–35)	31(26.5–35)	0.60
**Gestational age, weeks**	40.1 (39.2–40.5)	40 (38.5–40.5)	0.83
**Weight, Kg**	80.1(17.0)	79.8(16.2)	0.82
**Cervical dilation at AROM, cm**	3(2–4)	3(1.5–3)	< 0.01
**Cervical dilation at Oxytocin initiation, cm**	4(3–5)	3(2.5–4.2)	0.12
**Previous VBAC**	41(32%)	15(33%)	0.87
**No. of previous VBAC**	2(1–3)	2(1–3)	0.98
**Parity**	1(1–3)	2(1–4)	0.29

Numbers are n (%) or median (interquartile range) or mean (standard deviation)

The delivery outcomes are described in Tables 3 and 4. Among women who did not receive oxytocin, total time of IOL was significantly shorter (8.7 h vs.16.13 h *p* < 0.001). The timing of oxytocin initiation did not affect the total durance of oxytocin administration. (*p* = 0.96).Instrumental delivery did not differ between the groups.

**Table 3:** Delivery Outcomes for Women Who Received and Did not Receive Oxytocin after AROM

	**No oxytocin (492)**	**Oxytocin(173)**	***P***
**Birthweight, grams**	3332 (479.4)	3306(447.6)	0.61
**Total tome of IOL (from AROM to delivery), hours**	8.7 (9.3)	16.1 (12.3)	< 0.01
**Delivery mode**			
**SVD**	339(68.9%)	103(59.5%)	0.02
**Vacuum/forceps**	61(12.3%)	25(14.4%)	0.48
**Repeat CS**	92(18.6%)	45(26.0%)	0.04
**Successful VBAC**	400/492 (81.3%)	128/173 (73.9%)	0.03

Numbers are n (%) or mean (standard deviation)

**Table 4:** Delivery Outcomes for Women Who Received Oxytocin after AROM

	**Oxytocin started at < 12 h (128)**	**Oxytocin started after 12H (45)**	***P***
**Birthweight, grams**	3300 (451.7)	3325 (440.4)	0.29
**Total tome of IOL (from AROM to delivery), hours**	10.7(6.1)	31.3 (13.0)	< 0.01
**Total time of oxytocin administration, hours**	6.5(5.2)	8.0 (5.5)	0.96
**Time from AROM to oxytocin, hours**	3.5(2.1–6.1)	19.0(14.9–25.8)	< 0.01
**Delivery mode**			
** SVD**	78(60.9%)	25 (55.5%)	0.52
** Vacuum/forceps**	20(15.6%)	5 (11.1%)	0.45
** Repeat CS**	30(23.4%)	15 (33.3%)	0.19
** Successful VBAC**	98/128 (76.5%)	30/45 (67.6%)	0.19

Numbers are n (%) or median (interquartile range) or mean (standard deviation)

When comparing early vs. delayed administration of oxytocin there was no significant change in the VBAC rate (76.6% vs 67.5% *p* = 0.19). However the mean of total duration of the induction was 20.5 h shorter (10.8 h vs. 31.3 h *p* < 0.01) in the early group.

Women who received oxytocin were more likely to be diagnosed with chorioamnionitis compared to those who did not receive (18.4% vs 7.3%, respectively. *p* < 0.01). This risk did not differ when subdividing early vs late oxytocin administration (17.9% vs 20% *p* = 0.76). Two cases (0.4%) of uterine rupture occurred in the no oxytocin group, compared to none in the oxytocin group. There were two vs. one cases of low APGAR score (< 7) in no oxytocin and oxytocin groups, respectively (*p* = 0.77).

A regression analysis was preformed to identify factors associated with successful vaginal delivery. When adjusting for Age, number of previous births, cervical dilation prior to AROM and previous VBAC—oxytocin administration was not associated with higher rates of successful VBAC (aOR 1.26, CI 0.813- 1.972). In another model created to identify factors associated with chorioamnionitis, we found that oxytocin administration was associated with a decreased risk for chorioamnionitis (aOR 0.52. CI 0.304–0.917) even after adjusting for potential cofounders such as total time of IOL cervical dilation at AROM and history of previous VBAC.

A third model was created including solely women who received oxytocin, adjusting for potential confounders including maternal age, parity, VBAC history and cervical dilation at AROM. No association was found between timing of oxytocin administration and successful VBAC (aOR for late vs. early initiation- 2.78, 95%CI 0.74–10.31). Similarly, there was no association between timing of oxytocin administration and chorioamnionitis (aOR for late vs. early initiation-0.65, 95%CI 0.12- 3.38).

## Discussion

In this large cohort of women undergoing IOL for TOLAC, we found that for most women AROM alone was sufficient for induction of labor. However, in women requiring oxytocin, early initiation of oxytocin after AROM was associated with a shorter induction time without increasing rates of adverse maternal or neonatal outcomes. When comparing women who did or did not receive oxytocin, those induced by AROM alone had a higher rate of successful TOLAC, yet marked differences between these two groups have been found such as higher rate of previous vaginal delivery and prior VBAC, larger cervical dilation and older age. In a multivariable analysis controlling for these cofounders, we find no difference in the VBAC rates between the groups.

To date there is little information about the utility of AROM as a sole mode of IOL in women undergoing TOLAC. A recently published case–control secondary analysis of the MFMU (Maternal–Fetal Medicine Units Network) Cesarean Registry compared the prevalence of early AROM (4 < cm) in women with a successful vs failed TOLAC. They found that early AROM was associated with a 34% decrease in VBAC success (*p* < 0.01) with no higher rates of chorioamnionitis [19]. However, our study only includes women with early AROM, limiting the relevance of the MFMU study to our findings.

Our study suggests that AROM is an effective and safe mode of induction of labor for women with a previous CS. In the majority of such women, especially those who have had a prior VBAC, AROM alone is enough to lead to delivery without necessitating the use of oxytocin. Avoiding oxytocin use is advantageous in these women because of its increased risk of uterine rupture [3, 14, 15]. Notwithstanding, the average cervical dilation at presentation was relatively high in both groups, suggesting that our finding may be relevant mainly to patients with a ripened cervix.

In contrast to the advantages mentioned, women who received oxytocin had lower rates of chorioamnionitis, even after controlling for duration of IOL. A possible explanation for this finding is that women who developed signs of infection were transferred immediately to perform a cesarean section, and the induction process was halted.

When evaluating solely women who received oxytocin, we found there is no advantage of delaying oxytocin administration. Late onset of oxytocin resulted in a longer duration of induction with no difference in the rate of a successful VBAC.

Our study has several strengths. This is one of the few studies to evaluate AROM as a single mode of IOL in women with a previous CS. Furthermore, we had detailed demographic and clinical data available from our electronic medical record.

On the other hand, there are some limitations to consider. First, we were underpowered to assess rare outcomes including uterine rupture. Second, we did not have information on certain clinical characteristics including indication for the induction of labor and the reason for oxytocin administration. These missing details may have affected our findings since certain indications for IOL (e.g. gestational diabetes or fetal macrosomia) are associated with higher rates of cesarean section. Third, this is a single center study, limiting generalizability. Finally, as a retrospective observational study our findings may be influenced by confounding.

## Conclusion

AROM as a single mode of induction of labor in women with a previous cesarean section is a safe and efficient practice with high rates of successful VBAC. When spontaneous labor does not develop shortly after AROM, there is no advantage to delay the administration of oxytocin.

## Data Availability

The datasets used and/or analyzed during the current study are available from the corresponding author on reasonable request.

## Abbreviations

AROM: Artificical rupture of membranes
IOL: Induction of labor
TOLAC: Trial of labor after cesarean section
VBAC: Vaginal birth after cesarean section
